# Supplementary material for: Detecting differential transcript usage in complex diseases with SPIT
Source: bioRxiv. 2023 Jul 10:2023.07.10.548289. Preprint. [Version 1] doi: 10.1101/2023.07.10.548289 (PMC10369883; doi:10.1101/2023.07.10.548289)
Supplement: Supplement 2 [file media-2.docx]

**Supplementary Figure 1**

*Experiment 2*


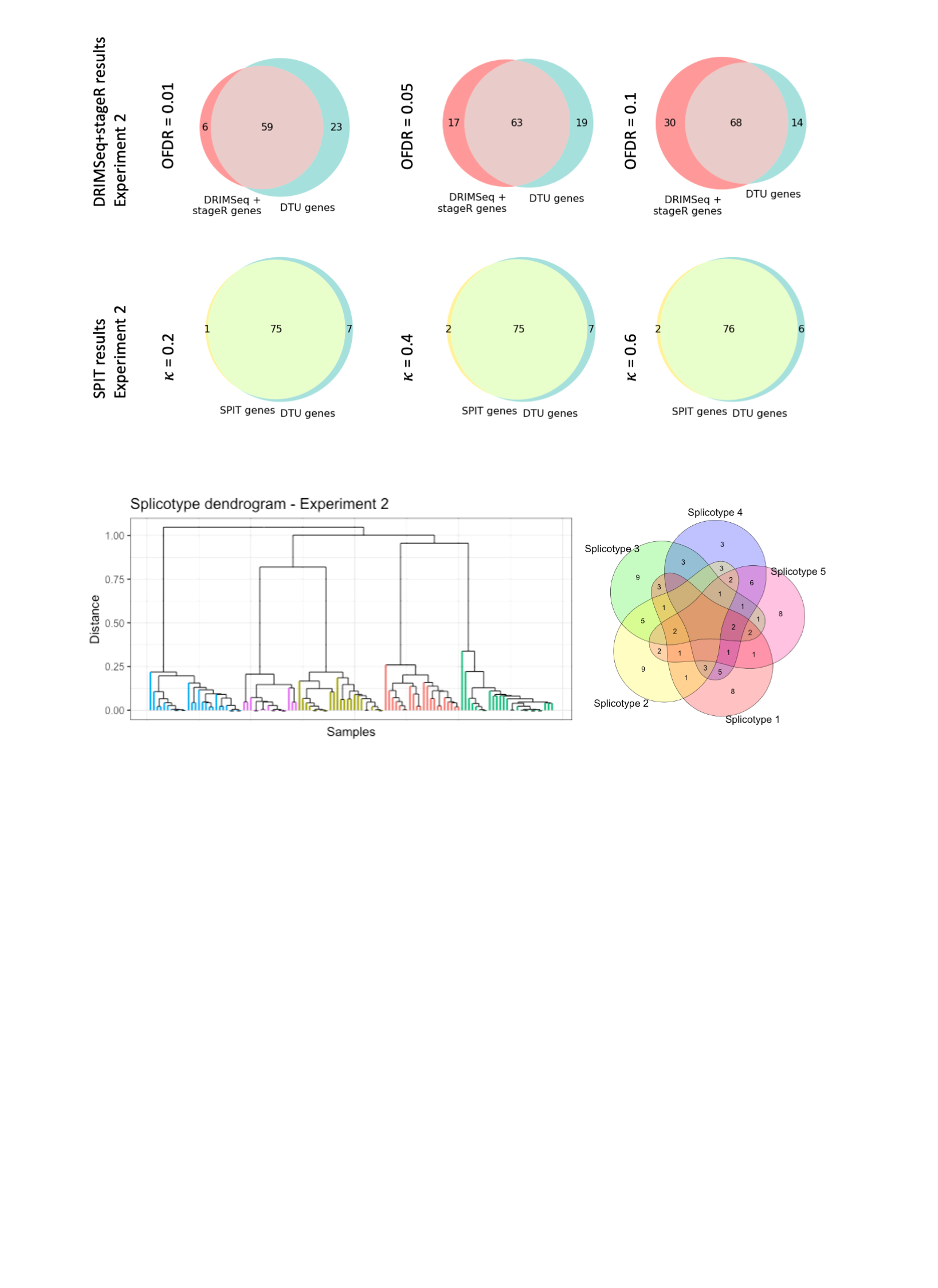


*Experiment 3*

**
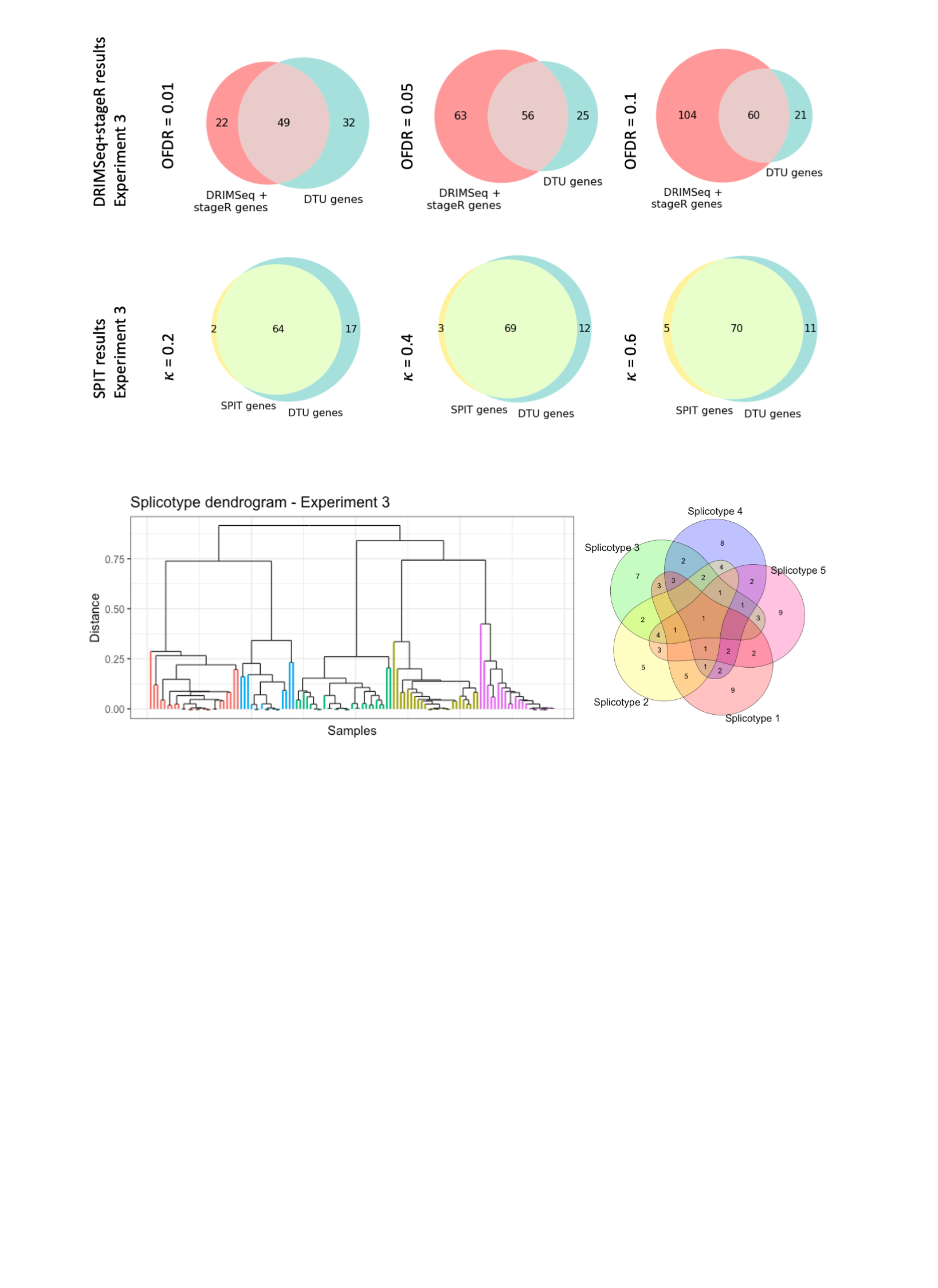
**

*Experiment 4*

**
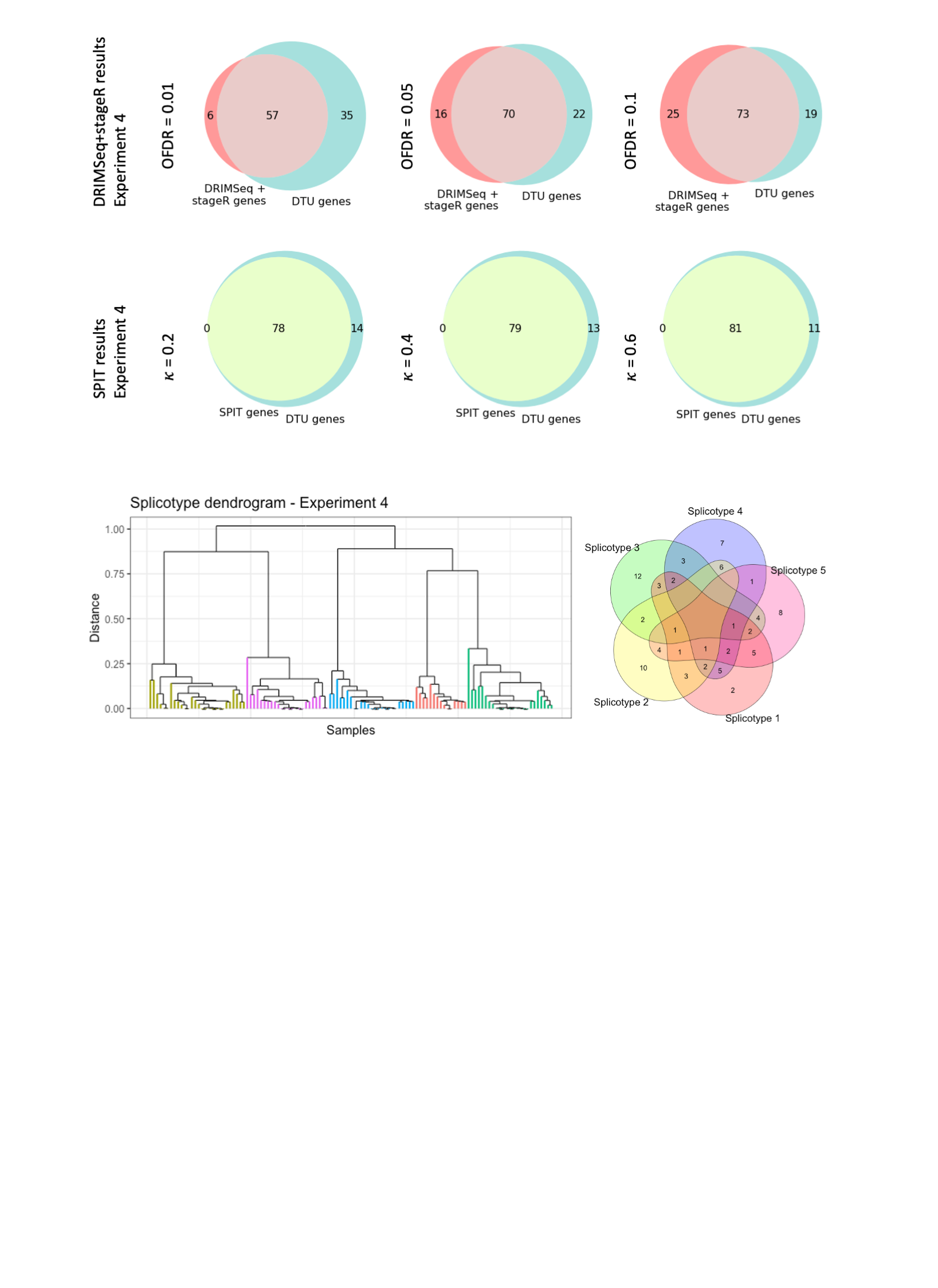
**

*Experiment 5*

**
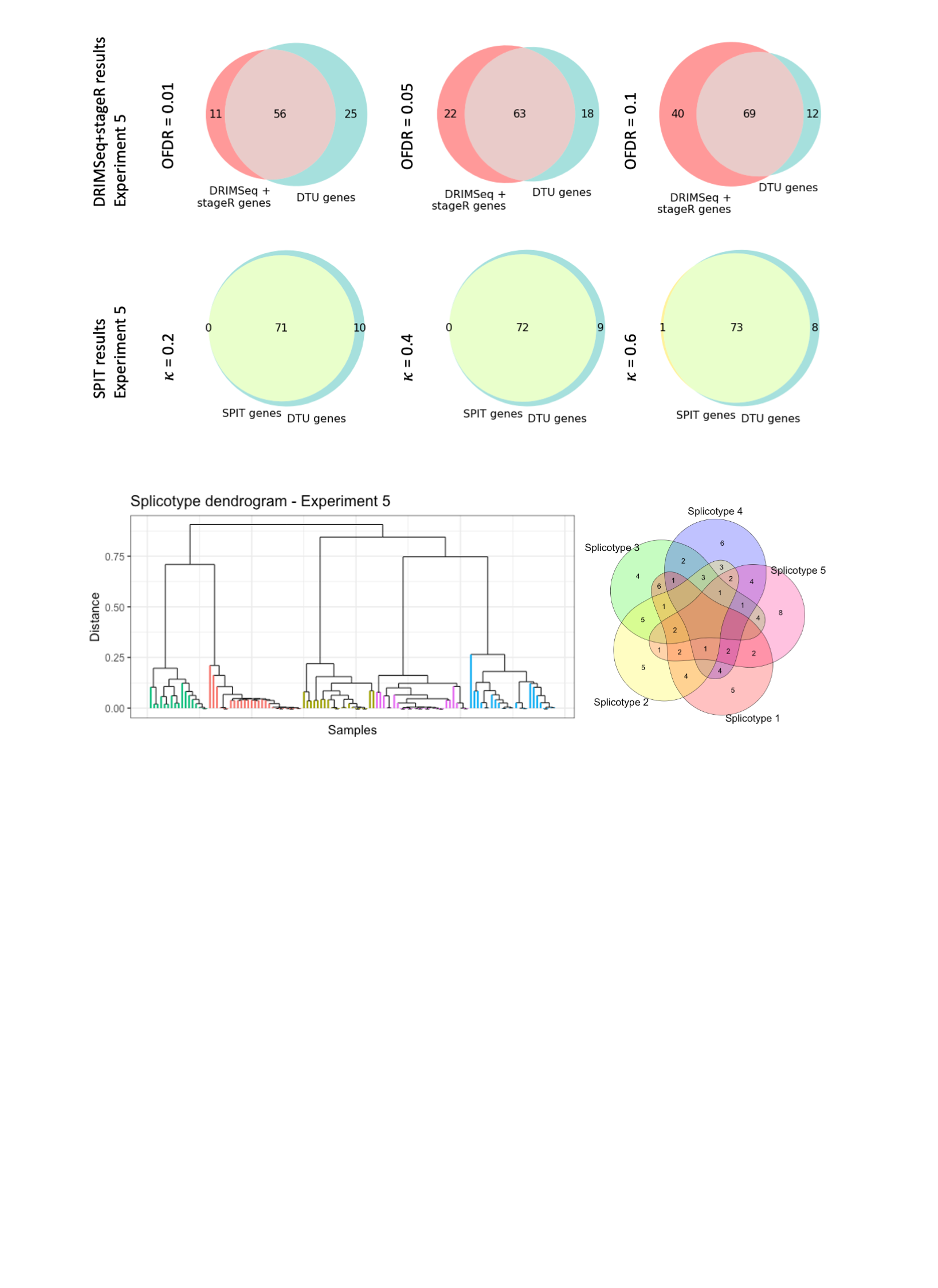
**

*Experiment 6*

*
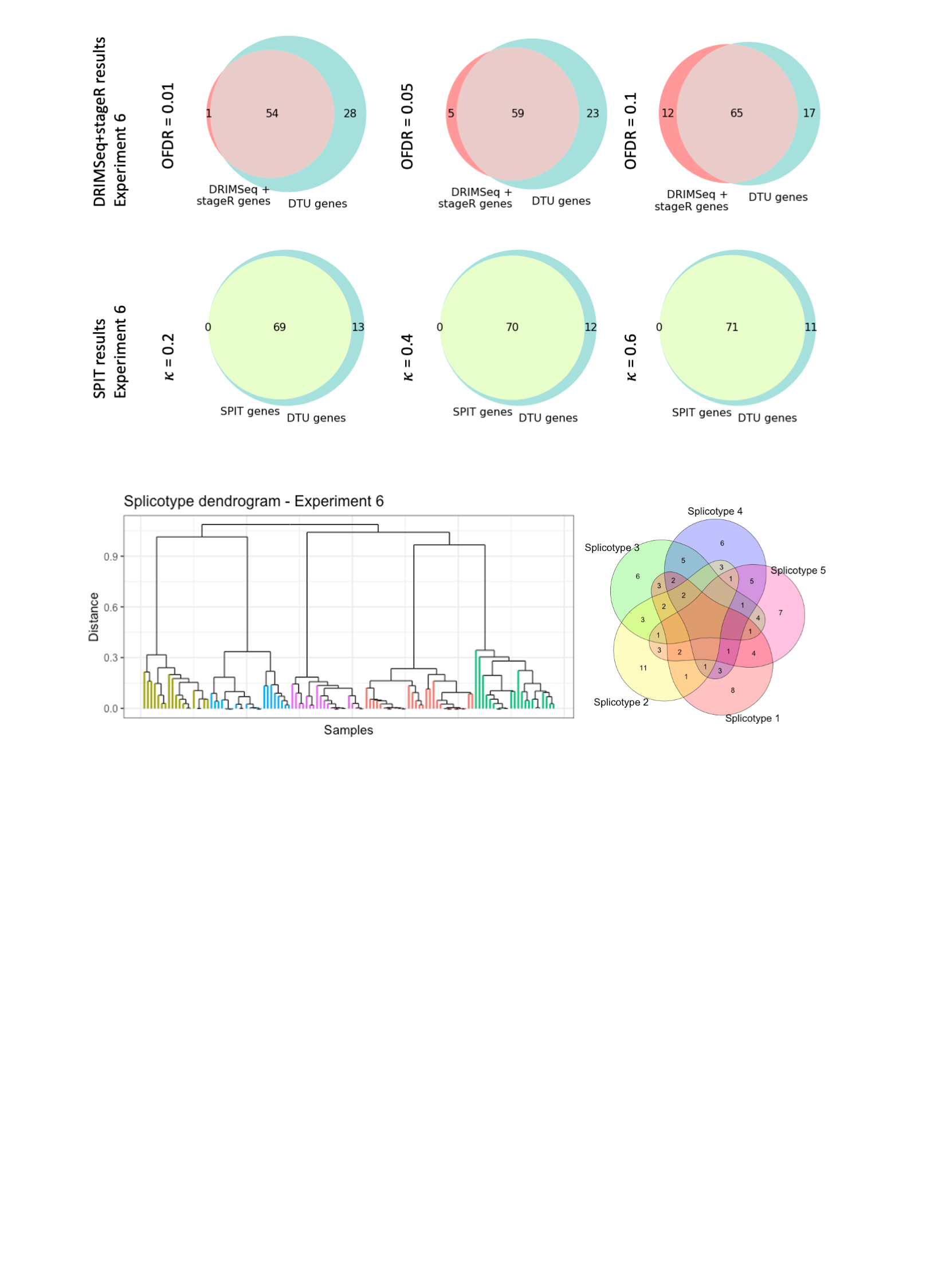
*

*Experiment 7*

*
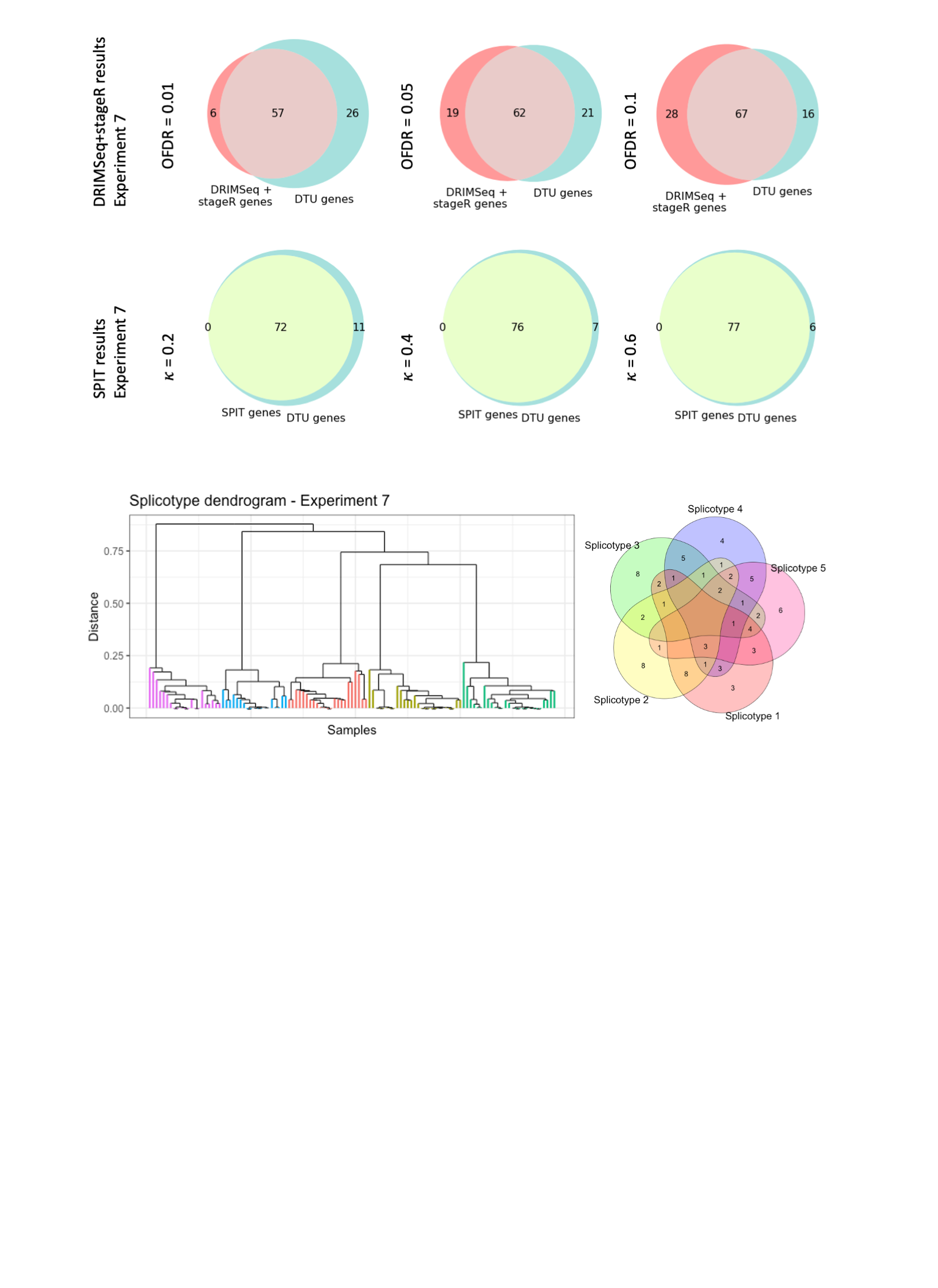
*

*Experiment 8*

**
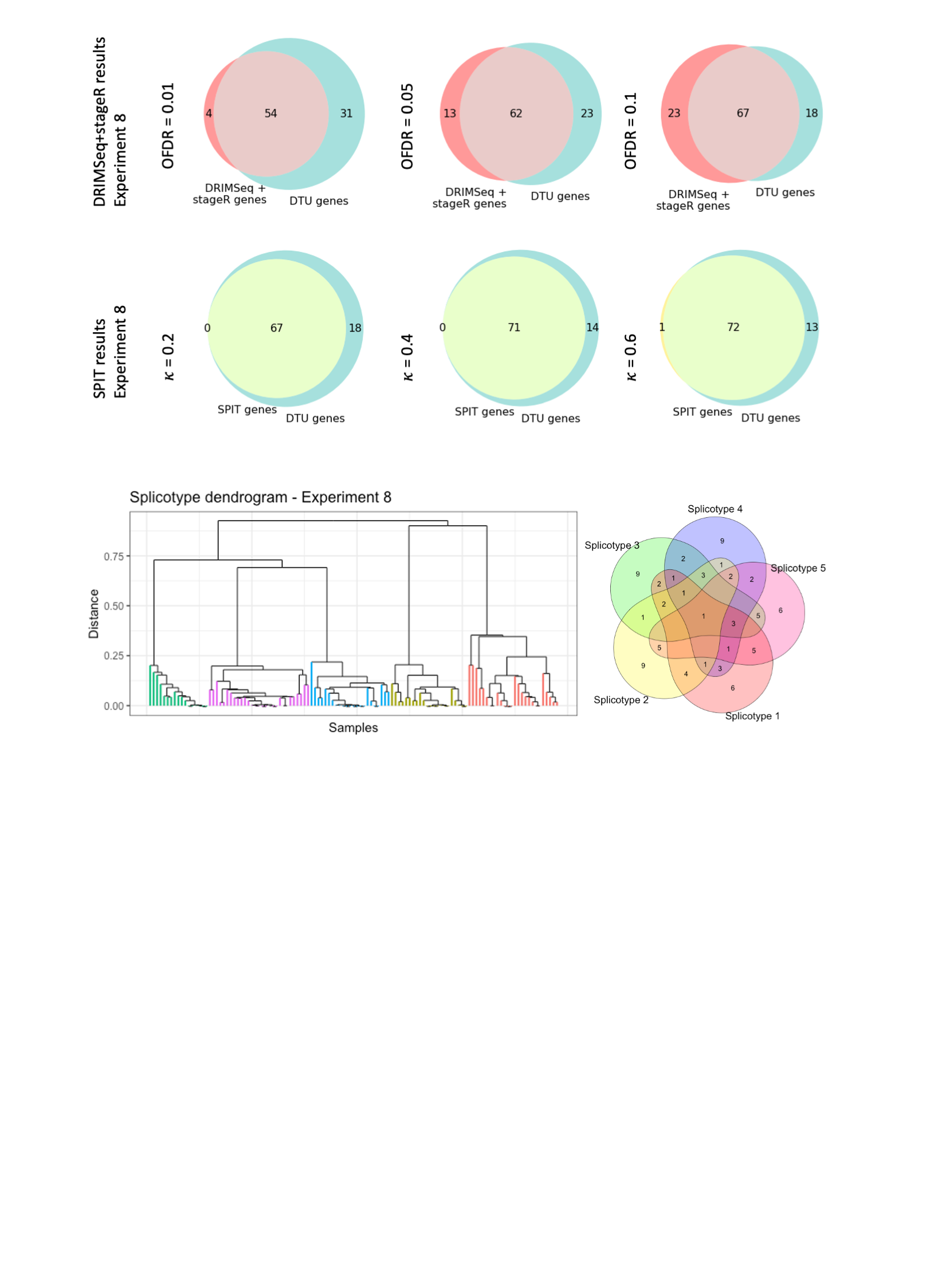
**

*Experiment 9*

*
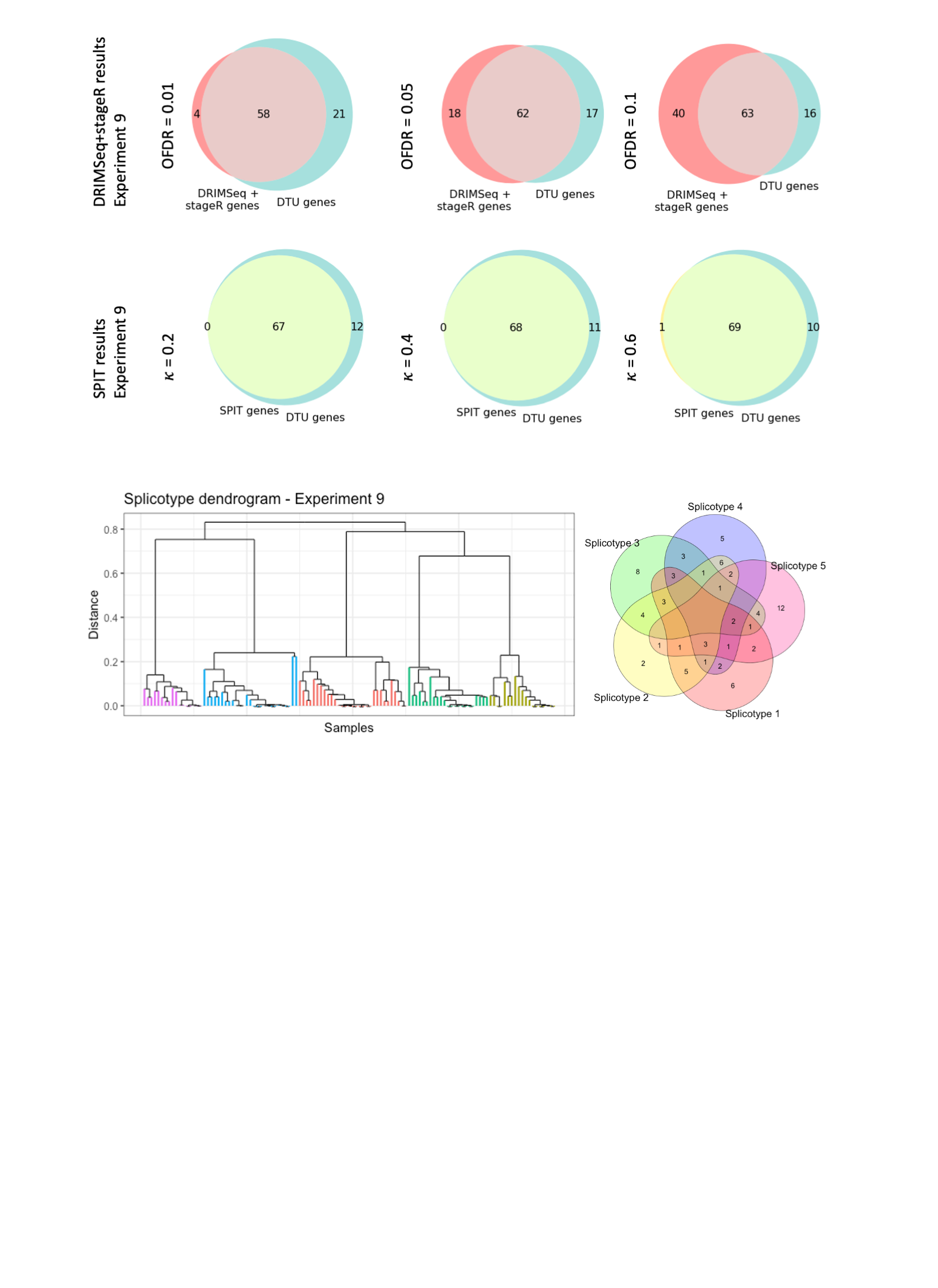
*

*Experiment 10*

**
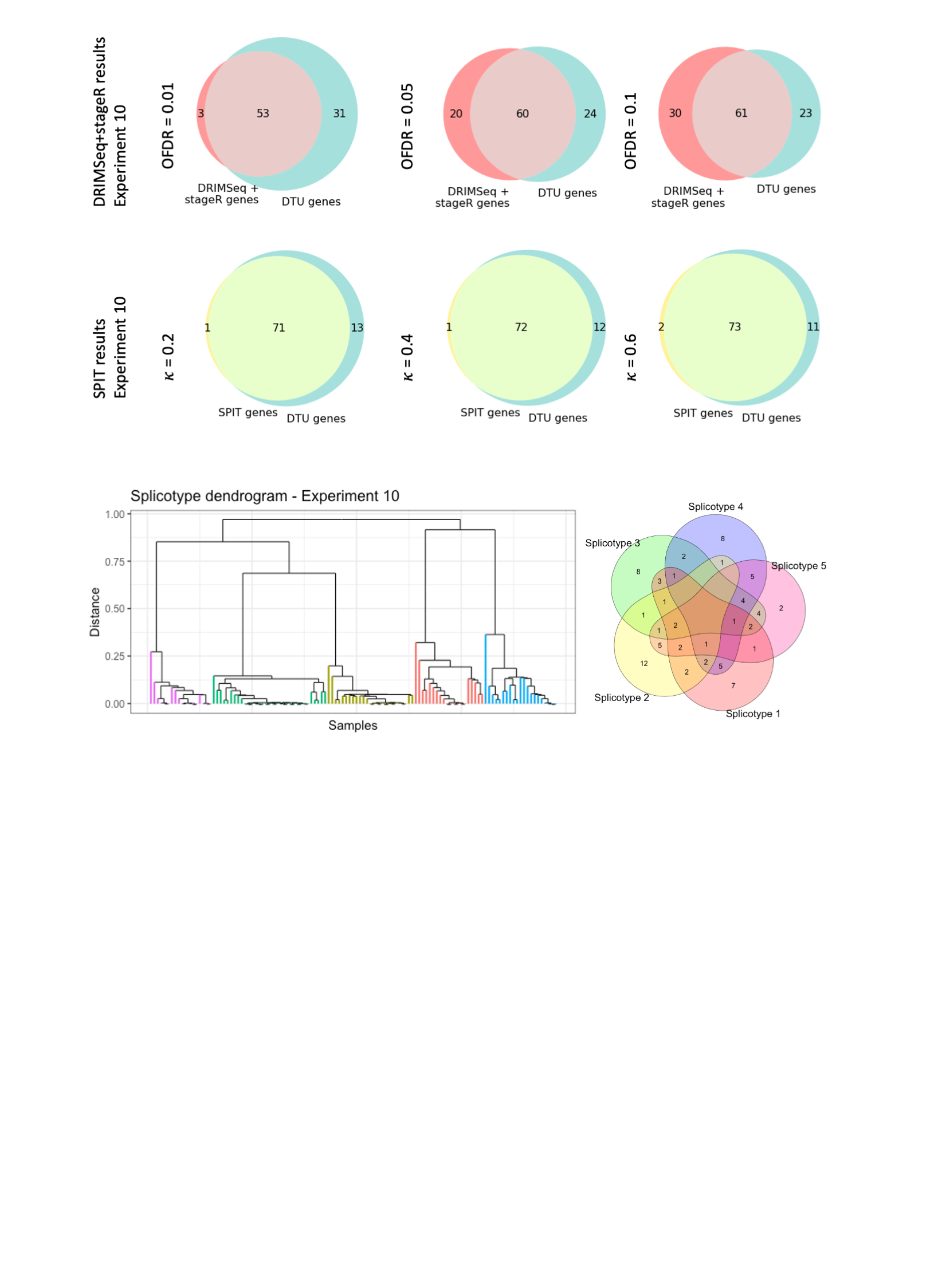
**

*Experiment 11*

*
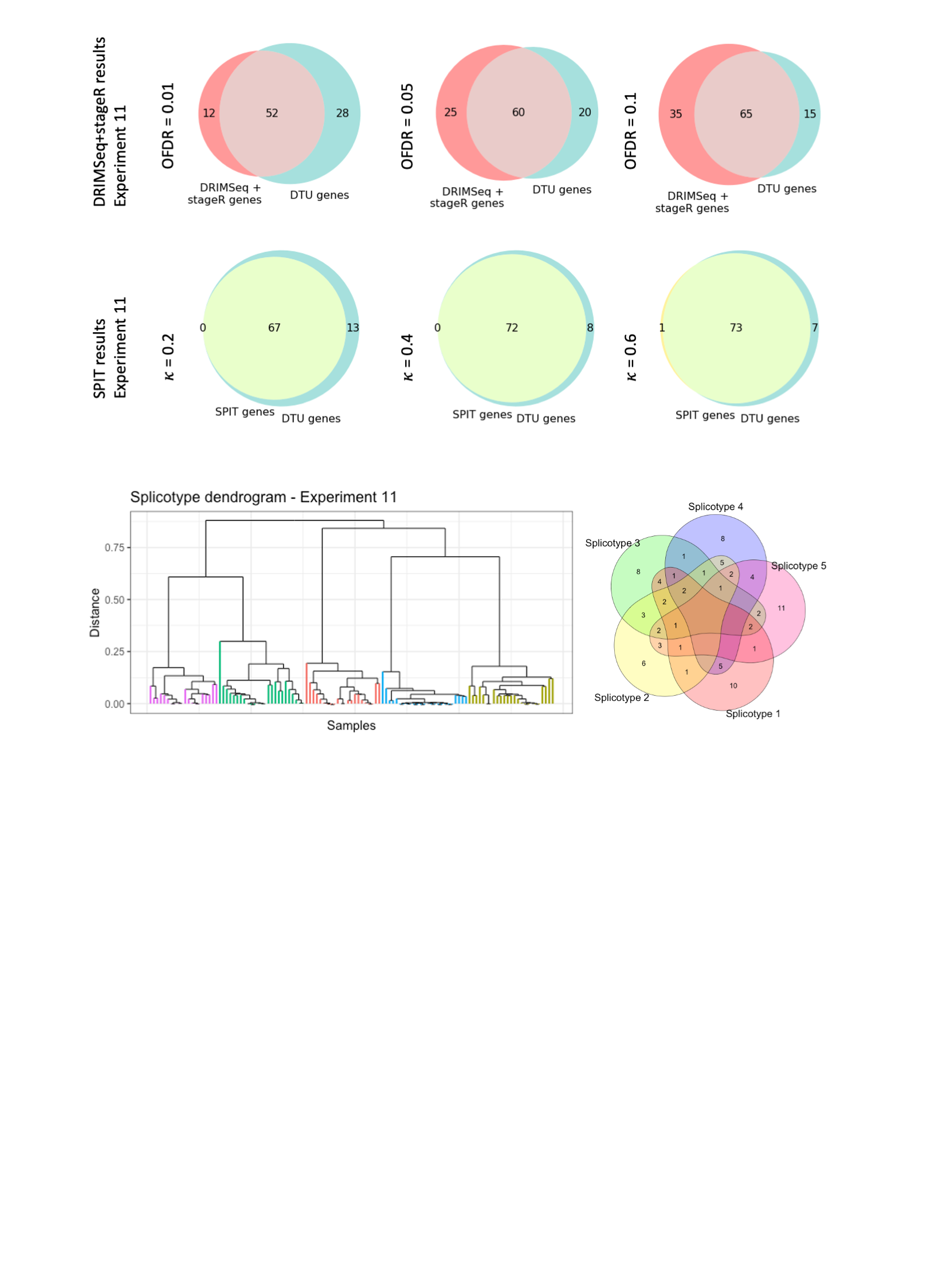
*

*Experiment 12*

**
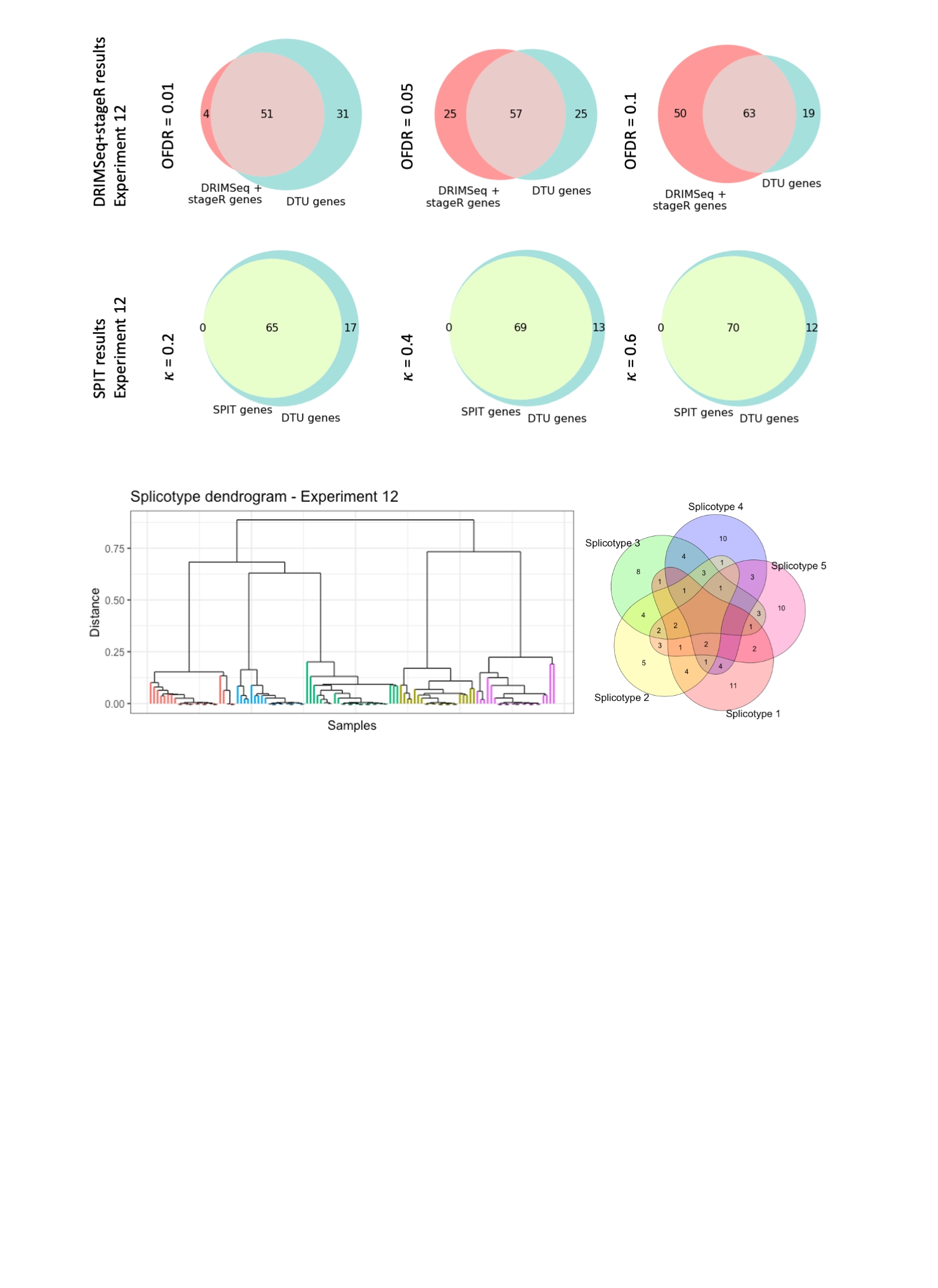
**

*Experiment 13*

**
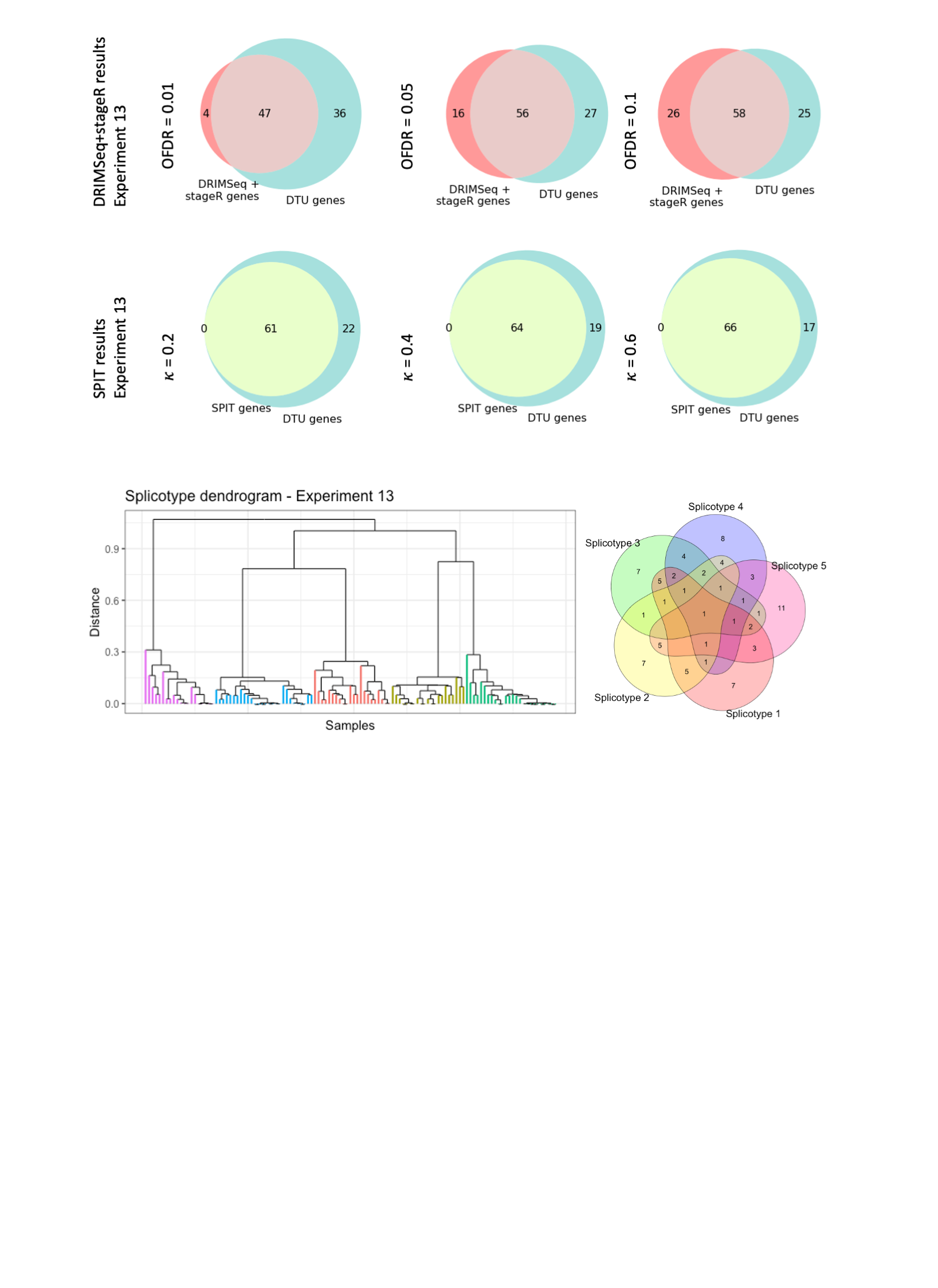
**

*Experiment 14*

**
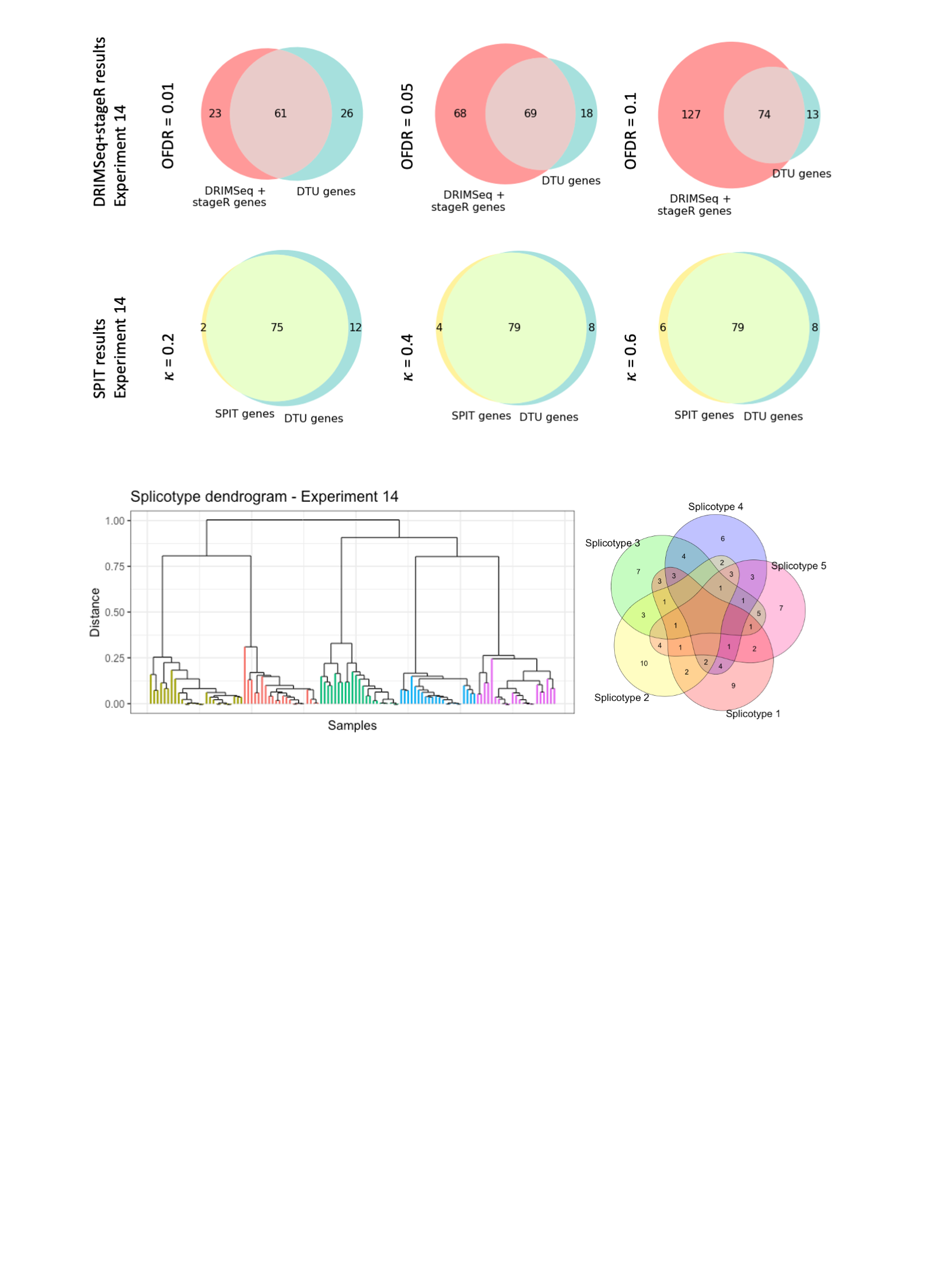
**

*Experiment 15*

*
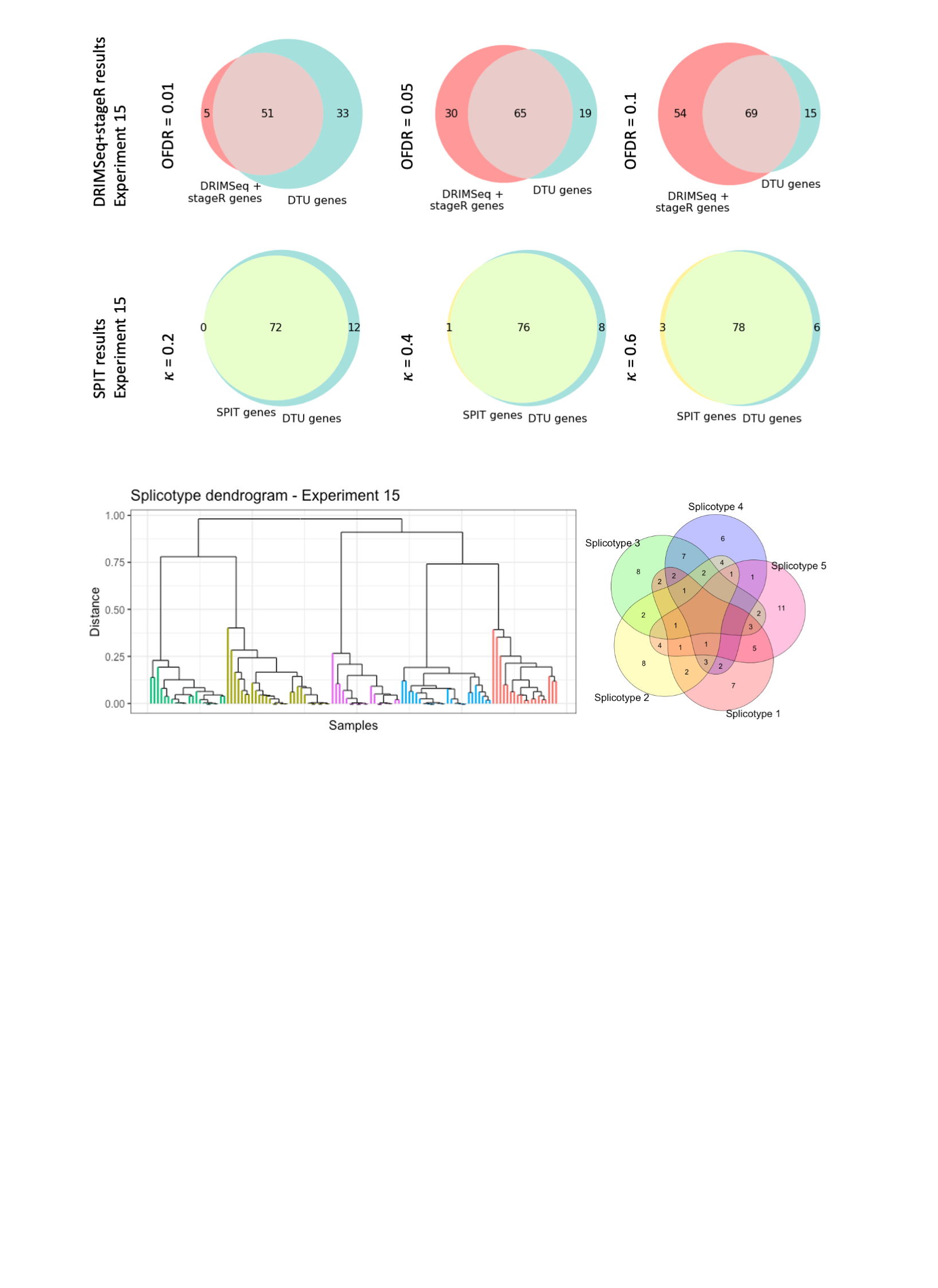
*

*Experiment 16*

*
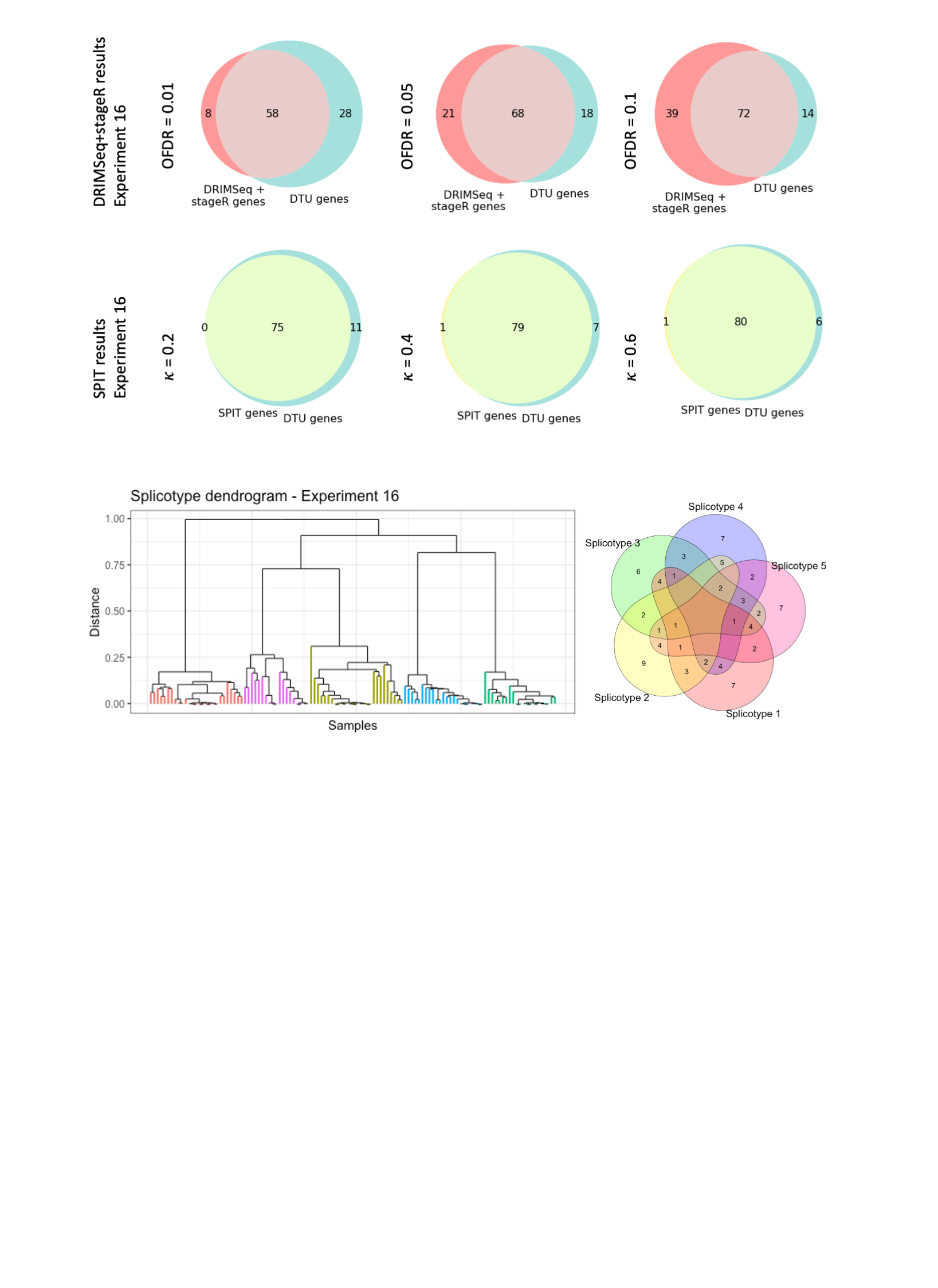
*

*Experiment 17*

*
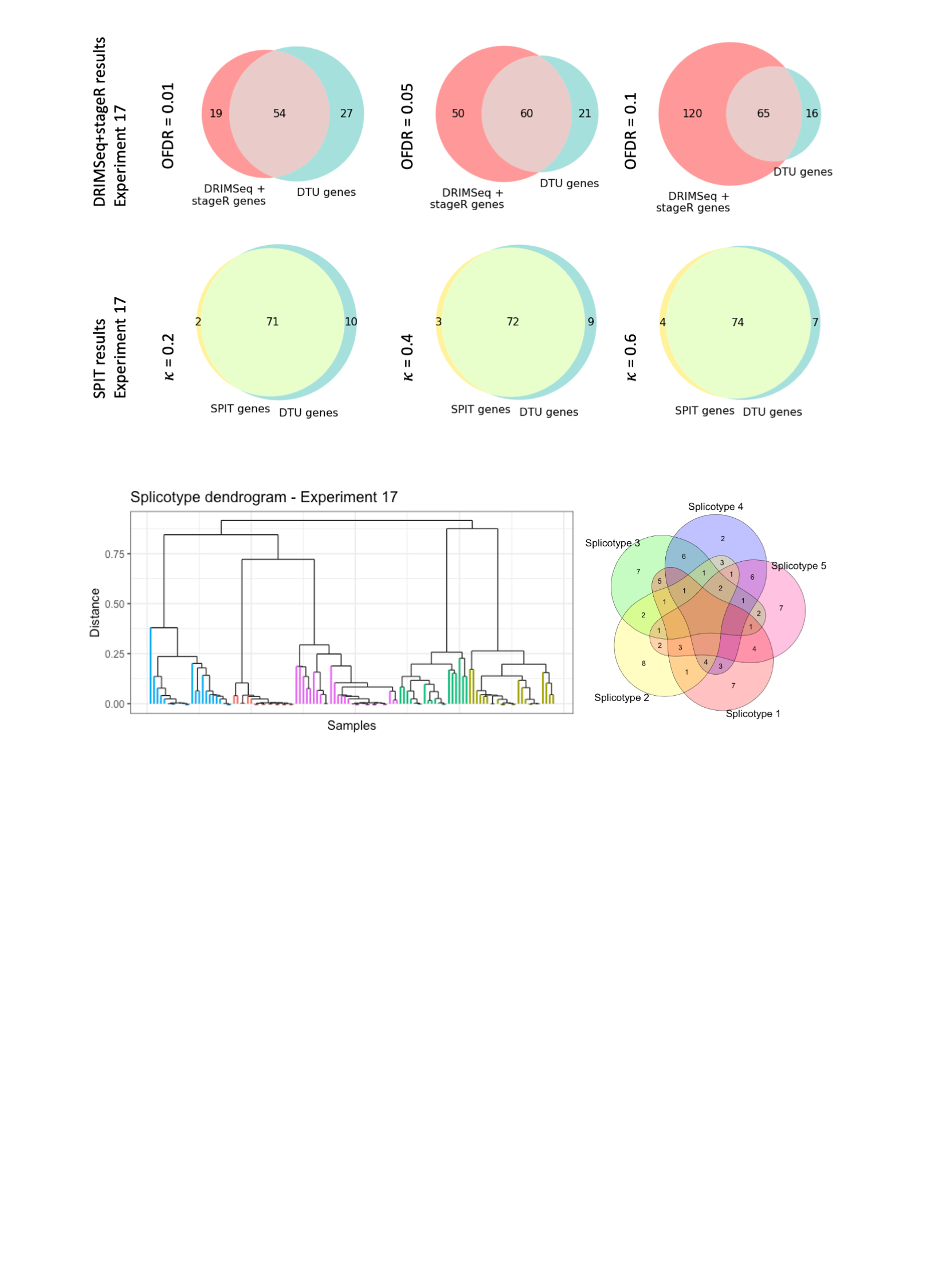
*

*Experiment 18*

**
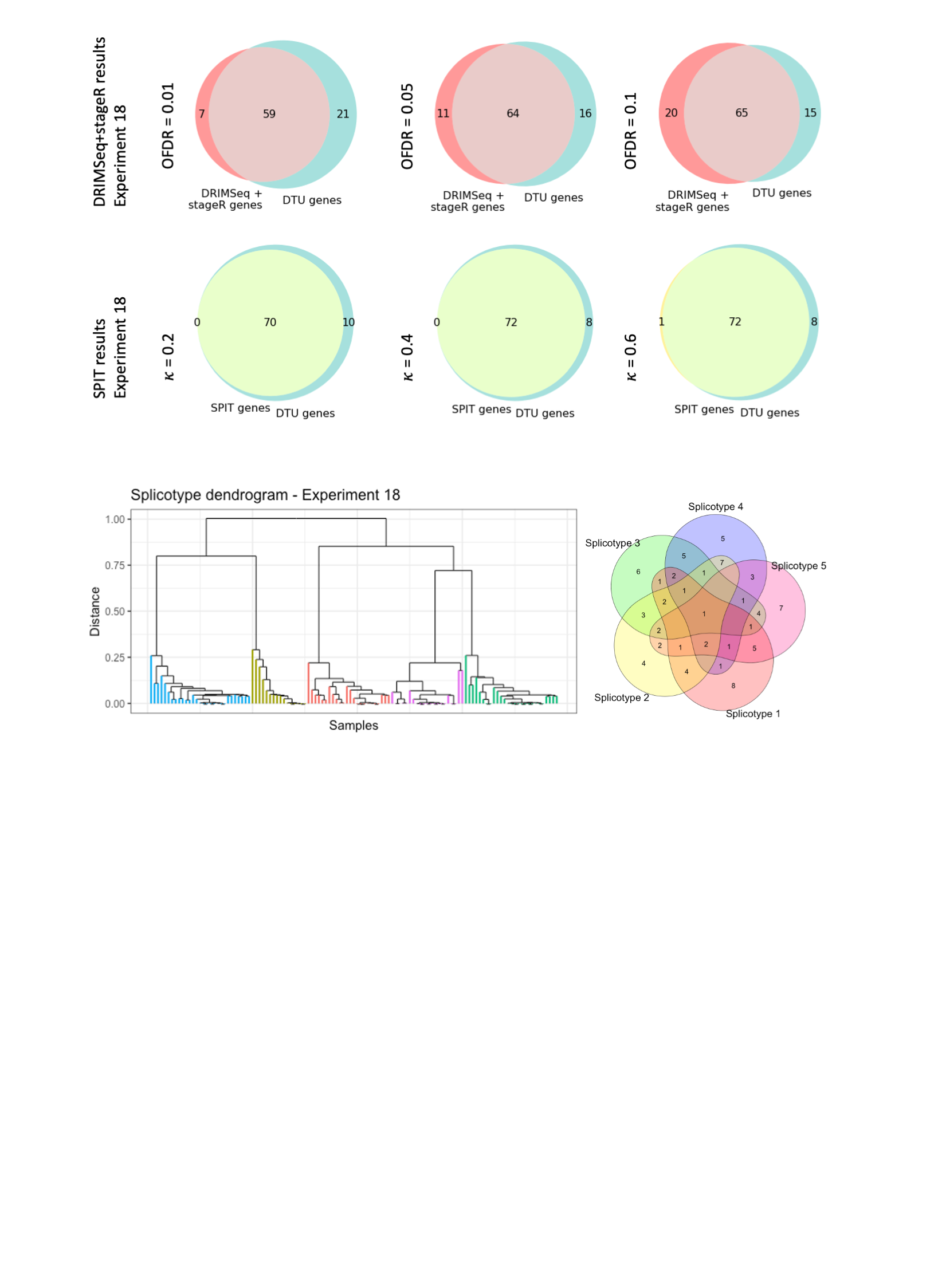
**

*Experiment 19*

**
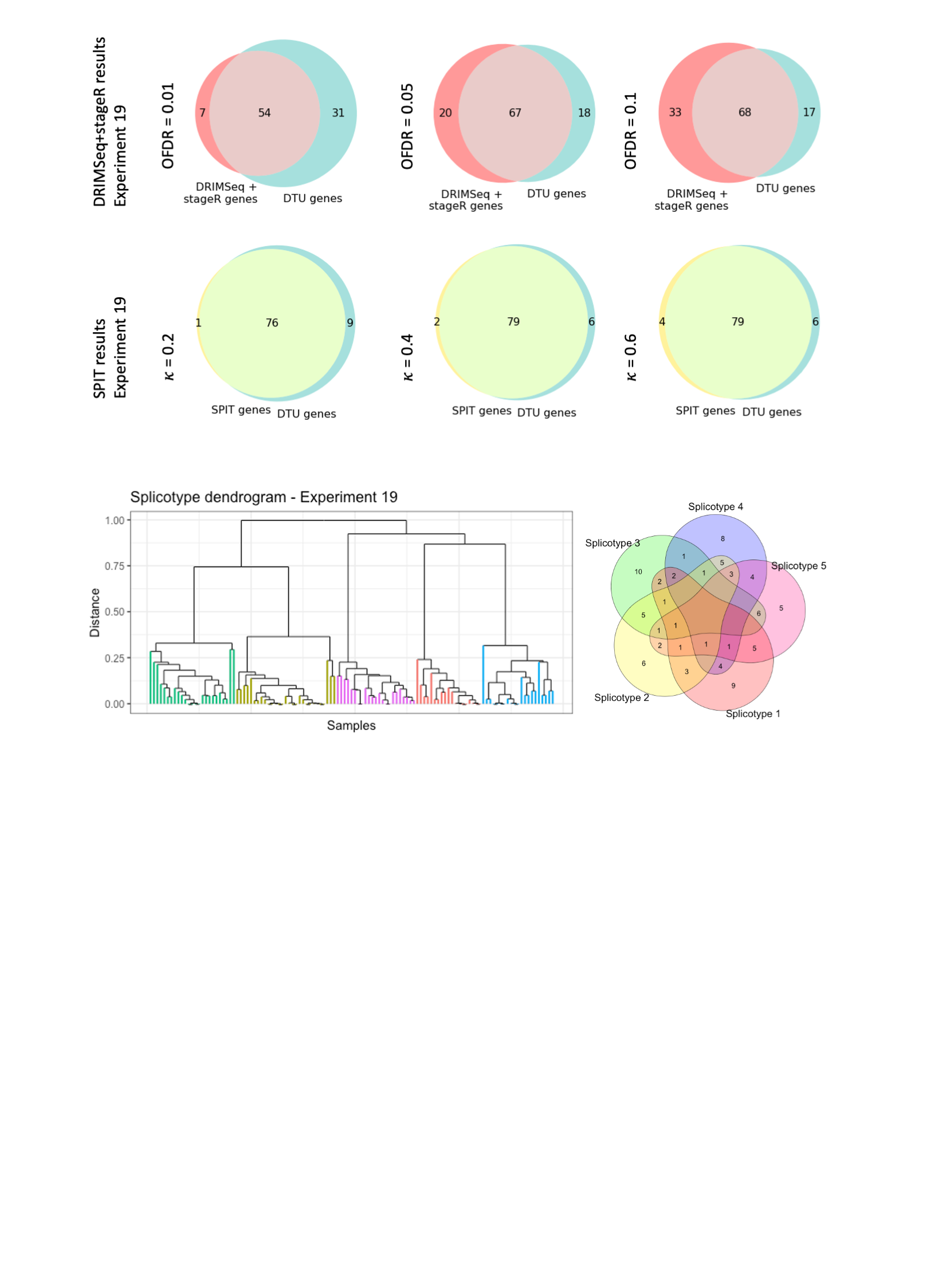
**

*Experiment 20*

**
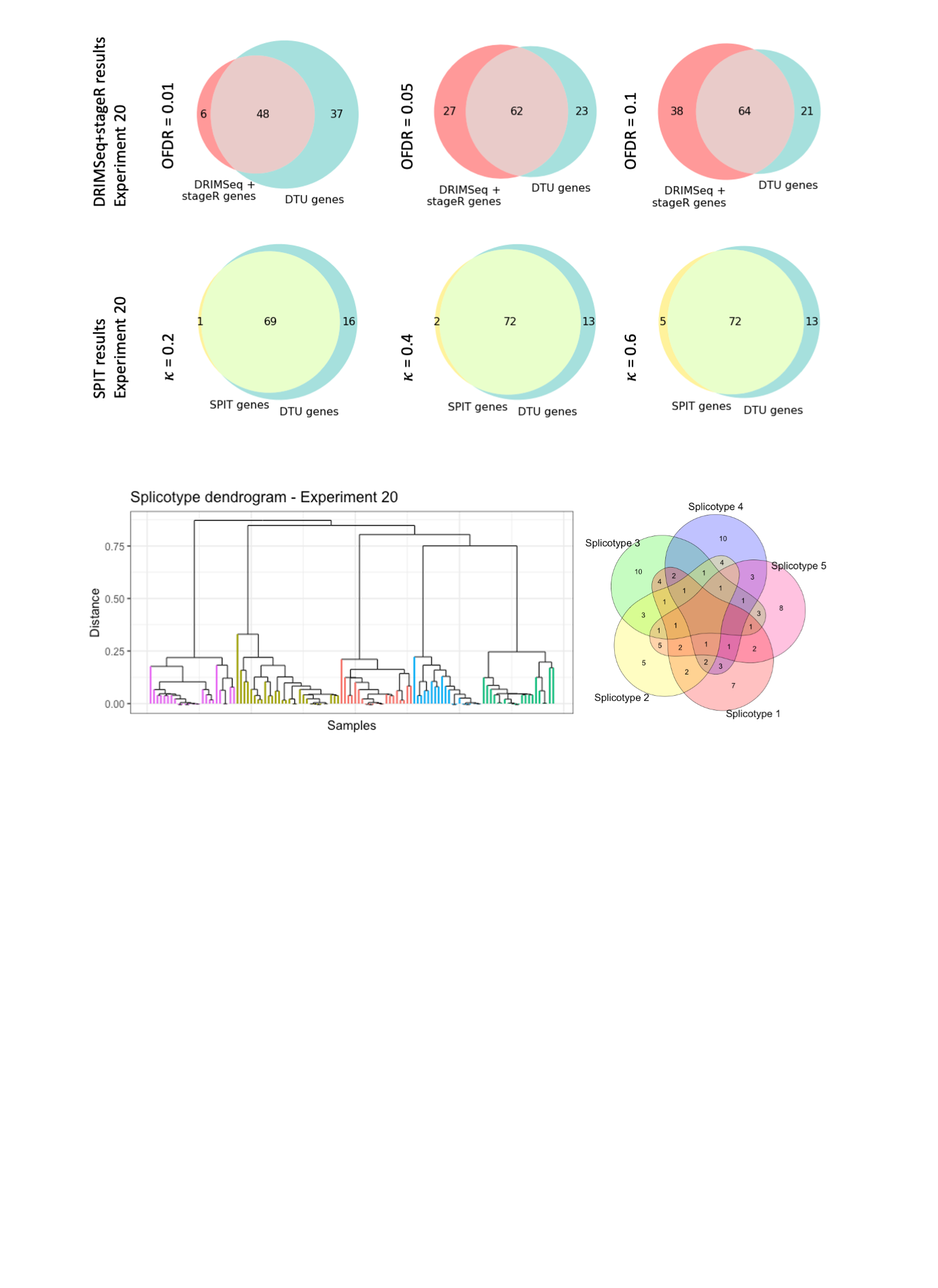
**

**Supplementary Figure 1:** Overlap of DRIMSeq+stageR pipeline and SPIT results with simulated DTU genes in experiments 2-19. The DTU event sharing Venn diagram and the corresponding final subcluster dendrogram based on the SPIT DTU matrix for experiments 2-19. The subclusters are color coded based on their distinct sets of simulated DTU events (splicotypes).

**Supplementary Figure 2**


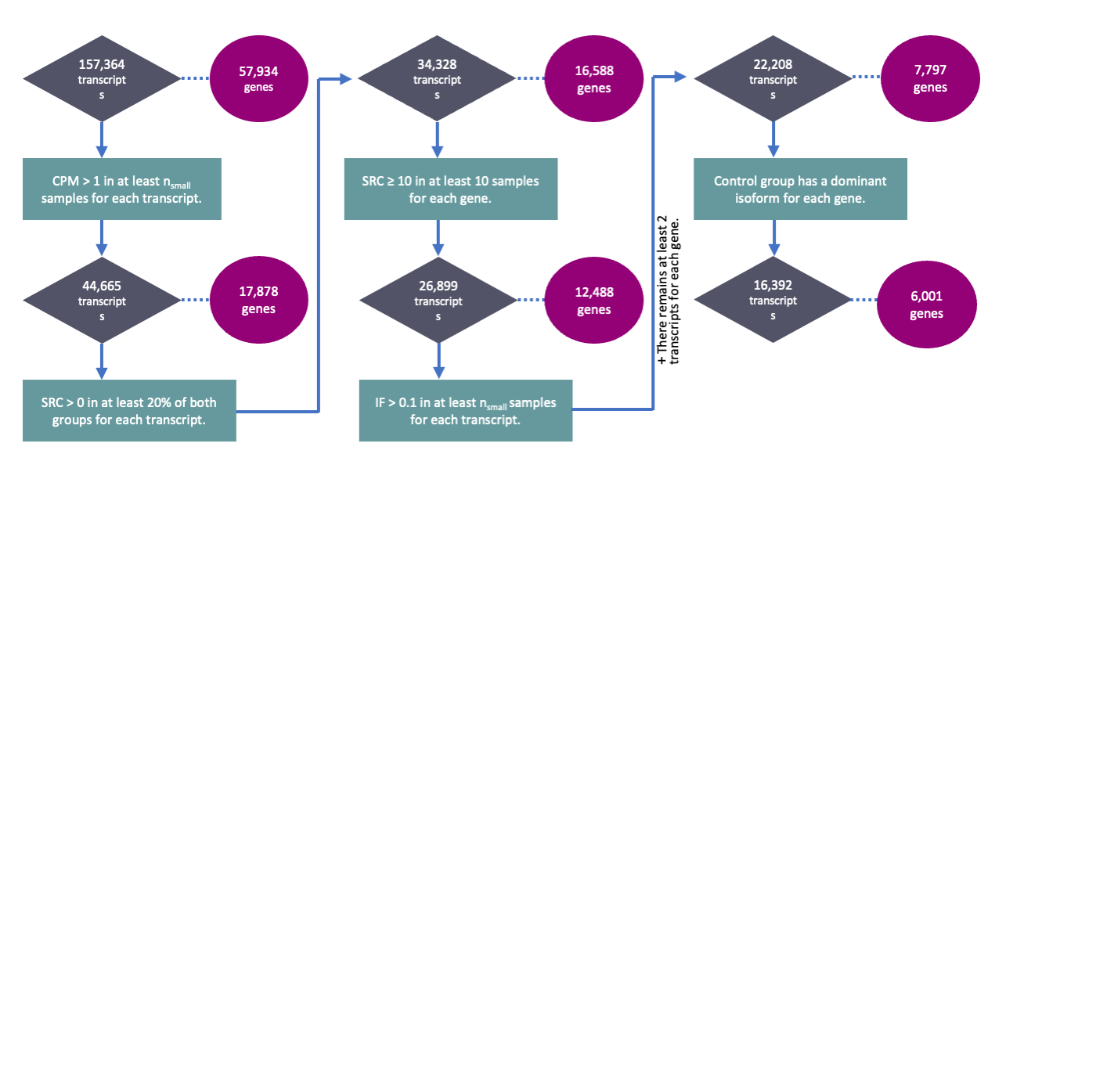


**Supplementary Figure 2:** Numbers of remaining transcripts and genes after each step of SPIT’s pre-filtering process applied on the Lieber brain samples with the default parameters.

**Supplementary Figure 3**

**
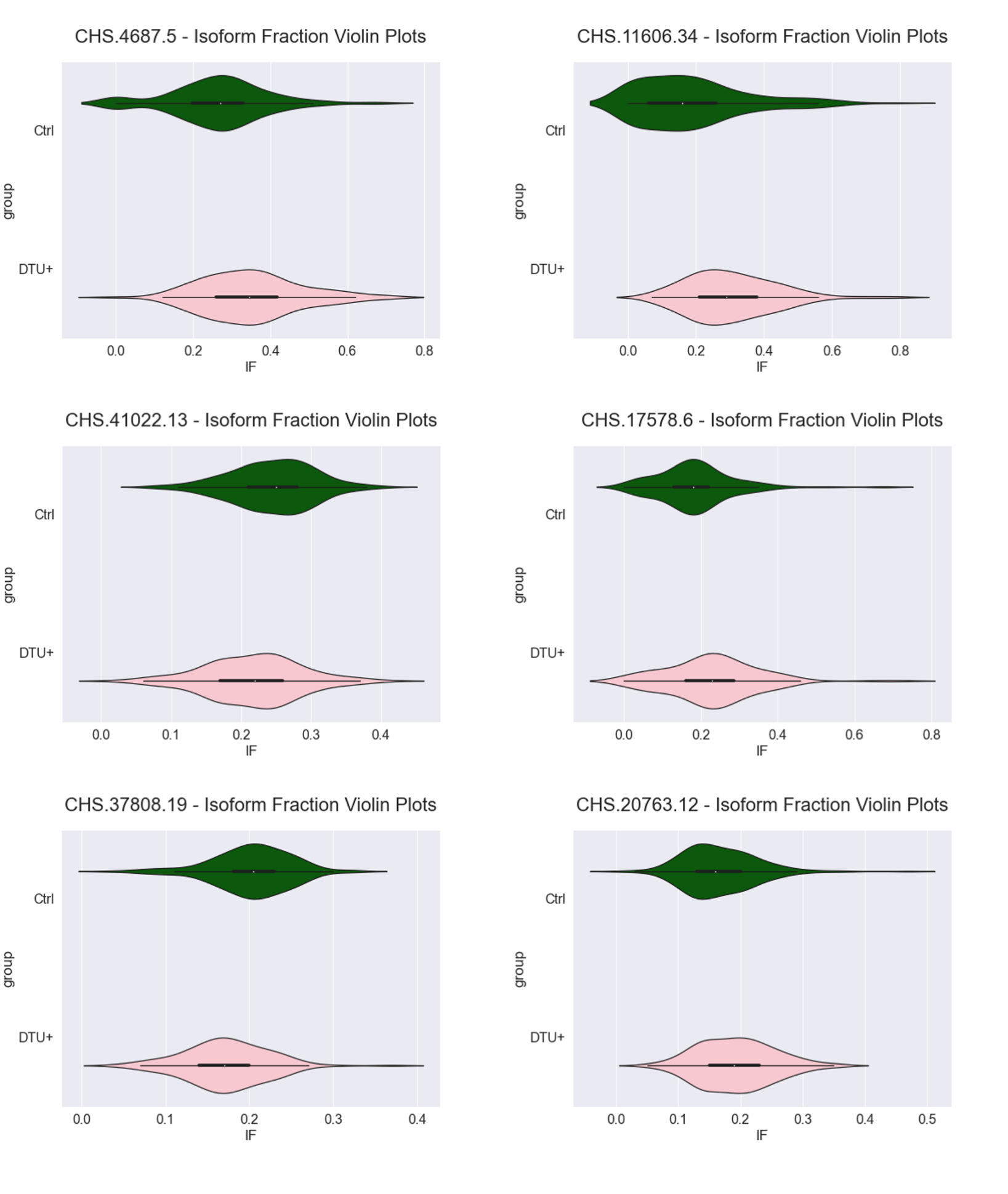
**

**Supplementary Figure 3:** Violin plots of the isoform fractions for the 6 transcripts of candidate DTU events in schizophrenia analysis.
