# Supplementary material for: Detecting differential transcript usage in complex diseases with SPIT
Source: bioRxiv. 2023 Jul 10:2023.07.10.548289. Preprint. [Version 1] doi: 10.1101/2023.07.10.548289 (PMC10369883; doi:10.1101/2023.07.10.548289)
Supplement: Supplement 3 [file media-3.pdf]

id	rin	condition	sex	race	age	batch
R12195	8.4	1	M	AA	62.61	2
R12198	8.5	1	M	CAUC	29.98	2
R12199	8.8	1	M	CAUC	65.26	2
R12200	7	1	M	AA	32.35	2
R12246	8.6	1	M	AA	52.18	2
R12258	8.6	0	F	CAUC	69.07	2
R12259	5.4	0	M	CAUC	48.1	2
R12260	7.3	0	M	AA	50.28	2
R12263	8.1	0	F	AA	47.01	2
R12264	8.4	0	M	CAUC	49.26	2
R12265	6.3	0	F	AA	35.21	2
R12266	8.5	0	M	CAUC	54.43	2
R12276	5.9	1	M	AA	75.22	2
R12277	5.4	0	F	AA	50	2
R12278	8.3	0	M	AA	24.79	2
R12279	8.1	0	M	AA	58.48	2
R12280	8.4	0	M	AA	22.09	2
R12281	7.9	1	F	AA	60.75	2
R12282	8.1	1	M	AA	47.35	2
R12283	8.5	1	M	AA	35.82	2
R12284	8.1	1	M	CAUC	25.59	2
R12285	6.3	0	F	AA	58	2
R12286	7.5	0	M	AA	40.39	2
R12287	8	0	F	AA	19.69	2
R12289	7.8	1	F	AA	61.93	2
R12290	8.4	0	M	AA	45.91	2
R12291	7.5	0	M	AA	18.05	2
R12293	8.3	1	F	AA	62.63	2
R12295	8.4	0	M	AA	41.55	2
R12296	8.3	1	M	AA	38.72	2
R12297	8.5	1	M	CAUC	43.38	2
R12298	7.1	0	M	AA	36.95	2
R12299	7.9	0	F	AA	30.48	2
R12300	6.9	0	F	AA	52.57	2
R12301	7.1	0	M	AA	57.89	2
R12302	6.1	0	M	AA	57.1	2
R12303	5.1	0	F	AA	48.42	2
R12304	7.9	1	M	AA	63.18	2
R12305	7.6	0	M	CAUC	63	2
R12306	6.3	1	F	AA	41.59	2
R12307	8.2	1	F	AA	49.65	2
R12308	7.7	0	M	CAUC	57.6	2
R12309	7.4	0	F	AA	57.59	2
R12310	6.6	0	F	AA	40.18	2

R12311	7.9	0	M	AA	58.48	2
R12312	8.7	0	M	AA	48.66	2
R12314	7.5	0	F	AA	59.58	2
R12316	7.8	1	F	AA	48.93	2
R12317	7.1	1	F	CAUC	71.81	2
R12318	7.9	0	M	AS	58.2	2
R12319	8	0	F	AA	67.73	2
R12320	9	1	M	AA	46.47	2
R12321	5.3	0	F	AA	44.14	2
R12322	8.2	0	M	AA	51.59	2
R12323	6.9	0	F	CAUC	51.91	2
R12324	7.8	0	F	CAUC	64.28	2
R12325	8.3	0	F	AA	36.48	2
R12326	6.8	0	M	AA	60.35	2
R12327	6.8	0	M	AA	68.54	2
R12328	8.7	1	F	CAUC	44.86	2
R12329	6.7	0	M	AA	32.98	2
R12330	7.5	0	M	AA	18.48	2
R12331	7.7	0	F	AA	53.86	2
R12332	7.6	0	M	AA	60.7	2
R12333	6.9	0	M	AA	73.23	2
R12334	7.5	0	F	AA	48.1	2
R12335	8.9	1	F	CAUC	26.07	2
R12336	7.3	0	F	AA	54.48	2
R12337	8.5	1	M	CAUC	24.33	2
R12338	7.6	0	M	CAUC	61.13	2
R12339	8	0	F	AA	41.17	2
R12340	6	0	M	AA	49.72	2
R12341	8.7	1	F	AA	48.26	2
R12342	7.1	0	M	CAUC	49.18	2
R12343	6.7	0	F	AA	51.8	2
R12344	5.7	0	F	CAUC	57.44	2
R12345	7.5	0	M	CAUC	46.53	2
R12346	7.2	0	M	AA	60.72	2
R12347	6.9	0	M	CAUC	18.41	2
R12348	6.8	1	F	AA	62.39	2
R12349	7.5	0	M	AA	58.19	2
R12350	6.8	0	M	CAUC	47.45	2
R12353	7.8	0	M	AA	62.74	2
R12355	8.6	1	F	AA	42.98	2
R12356	6.5	0	M	AA	58.4	2
R12357	8.1	0	F	AA	53.36	2
R12358	6.9	1	F	AA	63.05	2
R12359	7.4	1	F	AA	57.13	2
R12360	7.6	0	F	AA	38.59	2

R12361	6.8	0	F	AA	42.91	2
R12362	7.5	0	M	CAUC	24.61	2
R12363	7.8	0	M	CAUC	49.17	2
R12364	8.3	1	M	CAUC	30.86	2
R12365	7.9	1	M	AA	65.32	2
R12366	7.2	0	M	CAUC	41.01	2
R12367	8.4	1	M	CAUC	58.96	2
R12368	7.5	0	M	CAUC	28.35	2
R12369	8.2	1	F	AA	45	2
R12372	7.1	0	M	AA	51.55	2
R12373	8	1	M	CAUC	48.08	2
R12377	6.7	1	M	AA	53.24	2
R12378	6.8	1	M	AA	18.57	2
R12379	7.6	1	M	AA	21.54	2
R12380	8.1	0	F	HISP	56.09	2
R12381	8.3	0	M	CAUC	34.23	2
R12382	6.9	0	F	AA	40.82	2
R12383	5.2	1	F	AA	55.69	2
R12384	7	0	F	CAUC	38.39	2
R12386	7.3	1	M	AA	48.34	2
R12387	7.3	0	F	AA	65	2
R12389	7.2	1	F	CAUC	92.1	2
R12390	7.6	1	F	CAUC	96.92	2
R12391	7.6	1	F	AA	71.12	2
R12392	7.6	0	M	AA	64.52	2
R12393	6.9	1	F	AS	57.69	2
R12394	7.7	1	M	CAUC	30.97	2
R12395	8.1	1	M	AA	38.6	2
R12396	7.7	0	F	CAUC	30.02	2
R12398	7.5	0	F	AA	64.86	2
R12399	7.2	0	F	CAUC	19.26	2
R12400	10	0	F	AA	46.07	2
R12401	7.4	1	F	AA	62.68	2
R12402	5.5	1	F	AA	52.13	2
R12403	7.3	1	M	CAUC	70.24	2
R12404	10	0	M	CAUC	46.57	2
R12405	7.8	0	M	AA	53.26	2
R12406	5.4	0	F	CAUC	61.15	2
R12407	7.1	0	F	AA	54.56	2
R12408	7.8	0	M	CAUC	20.85	2
R12409	6.8	0	M	CAUC	51	2
R12410	6.7	0	M	CAUC	39.64	2
R12411	6.9	1	M	AA	57.58	2
R12412	7.4	1	M	AA	37.67	2
R12413	7.1	1	F	CAUC	56.91	2

R12414	7.9	0	M	CAUC	46.08	2
R12415	5.3	0	F	CAUC	33.97	2
R12416	5.3	1	M	AA	50.57	2
R12417	6	1	M	AA	83.41	2
R12418	6.5	1	M	CAUC	68.97	2
R12419	5.7	1	F	CAUC	54.53	2
R12420	6.3	1	F	CAUC	83.14	2
R12421	7.8	0	M	AA	27.2	2
R12422	7.5	1	M	AA	47.59	2
R12423	7.8	0	F	AA	53.56	2
R12424	7.6	0	M	CAUC	77.94	2
R12425	7.5	0	F	AA	71.77	2
R12426	7.9	1	M	CAUC	46.19	2
R12427	7.6	0	M	CAUC	46.94	2
R12428	7.6	0	M	CAUC	62.27	2
R12429	8.3	1	M	AA	43.05	2
R12430	8.1	1	M	AA	45.48	2
R12431	7.7	1	F	AA	34.84	2
R12432	8.4	1	M	HISP	48.81	2
R12433	7.7	0	F	CAUC	83.59	2
R12435	7.8	0	M	CAUC	42.81	2
R12436	7.8	1	M	CAUC	68	2
R12437	7.1	1	M	CAUC	42.71	2
R12438	8	1	M	CAUC	41.33	2
R12439	8.5	1	M	AA	52.02	2
R12440	7.4	1	M	HISP	79.32	2
R12441	7.5	1	M	AA	70.91	2
R12442	7.5	0	M	CAUC	63.2	2
R12443	6.8	0	M	CAUC	34.32	2
R12444	7.6	1	M	AA	48.51	2
R12445	8	1	M	AS	45.59	2
R12446	5.3	1	M	AA	53.18	2
R12447	7.6	0	F	CAUC	48.99	2
R12448	7.8	0	M	CAUC	56.05	2
R12449	7.2	0	M	AA	54.86	2
R12450	7.8	1	M	CAUC	22.1	2
R12456	8.3	0	M	AA	45.16	2
R12457	7.3	0	M	AA	38.48	2
R12458	8.6	0	M	AA	34.76	2
R12460	8.4	0	M	AA	46	2
R12461	7.8	0	M	AA	23.58	2
R12462	7.4	0	F	AA	46.71	2
R12463	7.1	0	M	AA	49.65	2
R12464	6.9	0	M	CAUC	52.49	2
R12465	8	1	F	CAUC	41.09	2

R12466	7.8	0	M	CAUC	55.51	2
R12470	7.9	0	M	AA	29.06	2
R12472	8.1	1	M	CAUC	62.2	2
R12473	6.6	1	M	AA	59.06	2
R12474	7.5	1	M	AA	50.34	2
R12475	6.9	1	F	CAUC	57.04	2
R12477	8	0	M	AA	73.86	2
R12478	7.3	1	M	CAUC	55.68	2
R12479	6.2	1	M	CAUC	56.15	2
R12480	7.3	0	M	CAUC	55.91	2
R12481	8.3	0	M	CAUC	40.08	2
R12482	7.1	0	M	AA	65.58	2
R12483	7.5	0	M	AA	40.24	2
R12485	7.8	0	M	AA	56.48	2
R12486	7.9	0	M	CAUC	36.53	2
R12487	5.4	0	M	CAUC	48.07	2
R12488	7.1	1	M	AA	49.26	2
R12489	6	1	M	AA	30.68	2
R12490	7.1	1	F	CAUC	50.73	2
R12491	7.1	0	M	CAUC	59.22	2
R12493	7.3	0	F	AA	40.31	2
R12494	6.1	0	M	AA	49.39	2
R12495	6.9	1	M	CAUC	50.8	2
R12496	7.9	0	M	CAUC	53.29	2
R12497	7.5	0	F	AA	50.84	2
R12498	7.5	0	M	CAUC	44.7	2
R12499	6.3	1	M	CAUC	20.34	2
R12501	6.2	0	M	AA	57.82	2
R12503	7.5	0	M	CAUC	63.5	2
R12505	5.1	0	F	CAUC	62.99	2
R12506	5.1	1	F	CAUC	59.93	2
R12508	7.5	1	M	CAUC	54.73	2
R12510	7.8	0	M	CAUC	23.22	2
R12511	7.8	0	F	AA	56.6	2
R12512	7.7	1	M	CAUC	38.41	2
R12515	6.8	1	F	AA	66.01	2
R12516	7.4	0	F	AA	35.8	2
R12517	6.7	0	M	CAUC	56.82	2
R12519	7.6	0	M	HISP	36.11	2
R12520	7.5	0	M	AS	27.74	2
R12521	6.8	0	F	AA	48.57	2
R12522	6.7	0	F	CAUC	60.51	2
R12523	6.7	1	M	CAUC	45.76	2
R12524	6.1	1	F	CAUC	47.56	2
R12525	7.6	0	M	CAUC	59.48	2

R12526	8	0	M	CAUC	46.12	2
R12527	6.9	1	M	AA	53.95	2
R12530	7.3	1	M	AA	53.74	2
R12531	7.7	0	M	CAUC	51.05	2
R12533	7.8	0	M	CAUC	52.33	2
R12535	7.2	0	M	AA	84.16	2
R12536	7.6	0	M	CAUC	54.9	2
R12537	8.6	0	M	AA	50.47	2
R12538	7.2	0	M	CAUC	40.38	2
R12539	7.3	0	M	CAUC	41.44	2
R12540	7.5	1	F	AA	33.35	2
R12551	8.2	0	M	CAUC	63.16	2
R12553	8.3	0	M	CAUC	42.05	2
R12554	8.3	0	M	CAUC	51.23	2
R12555	8.1	0	M	CAUC	51.63	2
R12557	8.1	0	F	CAUC	57.83	2
R12558	5.1	0	F	AA	50.6	2
R12560	6.9	0	M	CAUC	47.83	2
R12561	7.4	0	M	CAUC	72.43	2
R12562	8.2	0	M	AA	71.4	2
R12563	7	0	M	CAUC	61.75	2
R12564	7.4	0	M	HISP	33.28	2
R12565	8.1	0	F	CAUC	50.42	2
R12566	7.4	0	F	AA	43.71	2
R12567	8.2	0	M	CAUC	40.08	2
R12568	8.3	0	M	CAUC	34.56	2
R12569	8	0	F	CAUC	48.69	2
R12570	8.1	0	M	AA	34.57	2
R2816	8.4	1	F	AA	60.7	1
R2824	6.2	0	M	AS	33.22	1
R2867	8.5	1	F	AA	59.58	1
R2905	8.6	0	M	AA	22.58	1
R2947	8.7	0	M	AA	31.6	1
R3002	8.1	0	F	AA	26.77	1
R3029	8.3	0	M	HISP	48.47	1
R3030	8.3	1	F	AA	66.29	1
R3036	9	1	M	AA	75.57	1
R3050	7.2	0	M	AA	21.01	1
R3070	7.9	1	M	CAUC	81.16	1
R3393	8.4	0	M	AA	52.13	1
R3417	8.8	0	M	AA	19.89	1
R3456	8.9	0	M	AA	24.25	1
R3469	8.8	0	M	AA	45.86	1
R3470	8.8	0	M	AA	18.28	1
R3491	8.5	0	M	CAUC	32.67	1

R3496	8.9	1	M	CAUC	39.18	1
R3517	8.5	0	F	AA	42.68	1
R3540	8.2	1	M	AA	68.81	1
R3555	9	1	M	CAUC	66.81	1
R3557	9	0	M	CAUC	18.11	1
R3575	9.1	1	M	AA	37.3	1
R3597	8.7	0	M	AA	20.77	1
R3606	9.3	1	M	AA	35.46	1
R3611	8.8	1	M	AA	41.25	1
R3614	7.4	1	F	CAUC	60.08	1
R3616	8.8	1	M	CAUC	37.16	1
R3639	8.7	0	M	CAUC	49.53	1
R3645	5.9	1	F	AA	59.49	1
R3646	7.2	1	M	AA	49.35	1
R3665	8.4	0	M	CAUC	42.87	1
R3670	8.3	1	M	CAUC	20.22	1
R3774	8.8	0	M	CAUC	30.01	1
R3887	8.7	0	M	AA	22.16	1
R3888	6.9	1	F	AA	64.57	1
R3891	8.8	0	M	HISP	19.38	1
R3894	8.6	0	F	AA	31.53	1
R3900	6.8	1	F	CAUC	59.82	1
R3901	8.3	0	M	CAUC	50.87	1
R3907	8.6	0	F	AS	78.18	1
R3910	7.2	1	F	CAUC	32.01	1
R3927	8.1	0	F	AA	45.38	1
R3929	8.3	1	M	AA	60.11	1
R3932	8.7	0	M	CAUC	53.94	1
R3939	9.1	0	F	AA	29.95	1
R3944	7.9	0	M	AA	45.29	1
R3977	8.8	1	M	AA	60	1
R3978	7.6	1	M	CAUC	55.68	1
R4043	8.8	1	M	AA	51.71	1
R4046	8.2	1	M	CAUC	55.85	1
R4047	8.3	0	M	CAUC	33.24	1
R4108	9.4	1	M	AA	53.12	1
R4109	6.7	1	M	AA	74.56	1
R4111	8	1	M	AA	43.35	1
R4114	8.8	0	M	CAUC	28.57	1
R4162	5.9	1	M	AA	61.46	1
R4177	8.1	0	M	AA	22.71	1
R4179	8	0	M	AA	35.24	1
R4181	8	0	F	AA	21.53	1
R4194	8.3	0	M	HISP	20.21	1
R4200	7.8	0	M	AA	25.82	1

R4207	7	0	F	AA	44.82	1
R4226	8.9	0	M	AA	53.92	1
R4273	8.2	1	F	CAUC	28.22	1
R4279	9	0	M	CAUC	38.76	1
R4338	8.3	0	M	AS	70.9	1
R4339	8.4	1	M	CAUC	57.14	1
R4351	5.8	1	M	CAUC	52.13	1
R4355	7.4	1	M	CAUC	29.92	1
R4357	8.1	1	M	CAUC	45.94	1
R4364	8.3	1	M	CAUC	29.23	1
R4366	7	1	M	CAUC	49.4	1
R4367	7.2	1	F	CAUC	61.52	1
R4381	8.7	1	M	CAUC	34.06	1
R4383	8.5	1	F	CAUC	53.56	1
R4387	8.3	1	M	CAUC	28.59	1
R4388	6.4	1	F	AA	45.73	1
R4393	8.5	1	M	CAUC	21.24	1
R4407	8.5	1	F	CAUC	53.39	1
R4414	8.2	1	M	CAUC	18	1
R4417	7.8	1	F	AA	52.22	1
R5600	8.7	1	M	AA	44.64	1
R5608	7.1	1	F	CAUC	46.53	1
R5618	7.4	1	F	AA	66.94	1
R5622	8.7	0	M	AA	44.72	1
R5743	8.8	0	M	Multi-Racial	52.54	1
R5747	8.3	0	M	HISP	28.06	1
R5853	7.9	0	M	HISP	35.54	1
R5859	8.4	0	F	AA	21.32	1
R5861	8.6	0	M	AA	48.61	1
R5863	8	0	M	AA	49.73	1
R5868	7.7	0	M	AA	48.59	1
R5871	8.6	0	M	CAUC	18.72	1
R5885	8.2	0	M	CAUC	51.72	1
R5905	9.1	1	M	CAUC	53.97	1
R6531	9.1	1	M	AA	52.89	1
R6532	6.6	1	M	CAUC	19.69	1
R6534	6.7	1	M	CAUC	21.06	1
R6572	8.8	1	M	AA	70.52	1
R6578	8.4	1	M	CAUC	57.58	1
R6579	8.9	1	M	CAUC	60.56	1
